# Mutation in Parkinson Disease-Associated, G-Protein-Coupled Receptor 37 (GPR37/PaelR) Is Related to Autism Spectrum Disorder

**DOI:** 10.1371/journal.pone.0051155

**Published:** 2012-12-12

**Authors:** Eriko Fujita-Jimbo, Zhi-Ling Yu, Hong Li, Takanori Yamagata, Masato Mori, Takashi Momoi, Mariko Y. Momoi

**Affiliations:** 1 Department of Pediatrics, Jichi Medical University, Yakushiji, Shimotsukeshi, Tochigi, Japan; 2 Department of Pediatrics, Shengjing Hospital of China Medical University, Shenyang, Liaoning, China; 3 Department of Pediatrics, Detroit Medical Center, Michigan, United States of America; 4 Center for Medical Science, International University of Health and Welfare, Kitakanemaru, Ohtawara, Tochigi, Japan; Emory University, United States of America

## Abstract

Little is known about the molecular pathogenesis of Autism spectrum disorder (ASD), a neurodevelopmental disorder. Here we identified two mutations in the G-protein-coupled receptor 37 gene (*GPR37*) localized on chromosome 7q31–33, called the *AUTS1* region, of ASD patients; 1585–1587 ttc del (Del312F) in one Japanese patient and G2324A (R558Q) in one Caucasian patient. The Del312F was located in the conserved transmembrane domain, and the R558Q was located in a conserved region just distal to the last transmembrane domain. In addition, a potential ASD-related GPR37 variant, T589M, was found in 7 affected Caucasian men from five different families. Our results suggested that some alleles in *GPR37* were related to the deleterious effect of ASD. GPR37 is associated with the dopamine transporter to modulate dopamine uptake, and regulates behavioral responses to dopaminergic drugs. Thus, dopaminergic neurons may be involved in the ASD. However, we also detected the Del321F mutation in the patient's unaffected father and R558Q in not only an affected brother but also an unaffected mother. The identification of unaffected parents that carried the mutated alleles suggested that the manifestation of ASD was also influenced by factors other than these mutations, including endoplasmic reticulum stress of the mutated proteins or gender. Our study will provide the new insight into the molecular pathogenesis of ASD.

## Introduction

Autism spectrum disorder (ASD) is a neurodevelopmental disorder characterized by impaired communication and social interactions, restricted interests, and repetitive behavior. To date, several genes, including *NLGN 3* and *4*, *CADM1*, *CNTNAP2*, and *SHANK3* [1]–[5], were shown to confer susceptibility to ASD. Additionally, *TSC1/2* mutations are frequently associated with autism [6], [7]. However, little is known about how these genes are involved in the molecular pathogenesis of ASD.

One potential molecular pathogenetic mechanism is an imbalance between inhibitory and excitatory receptors. GABAergic functions are altered in mice with the autism-related mutation, *NLGN3* (R451C) [8]. GABRB2 levels were altered in mice with *CADM1* knocked out (submitted). G-protein-coupled receptors (GPCRs) are composed of seven transmembrane domains. GPCRs play important roles in a variety of biological processes that involve hormone receptors, neurotransmitter receptors, including GABA receptors, and other receptors [9]. Most G-proteins and GPCRs are expressed in the brain. They play important roles in brain function and neural development by mediating synaptic transduction and synaptic formation, respectively [10]. Furthermore, alterations of GPCR signaling are involved in a variety of neurological disorders, including dysmorphogenesis, psychosis, and/or mental disabilities. The majority of genes responsible for mental retardation are G-proteins, GPCRs, or genes linked to GPCRs or small G proteins [11]–[17].

We focused on *GPR37*, because it is localized on chromosome 7q31–33, called the *AUTS1* region, to indicate the first locus linked to autism [18]–[20]. GPR37 is associated with Parkinson's disease; it associates with the dopamine transporter to modulate dopamine uptake, and it regulates behavioral responses to dopaminergic drugs. It is also involved in dopamine metabolism in the nigrostriatal system. A deficiency in this receptor induces abnormal behavior, with enhanced sensitivity to amphetamine [21].

Here, we analyzed *GPR37* in patients with autism to identify mutations related to ASD.

## Results

**Figure 1:**
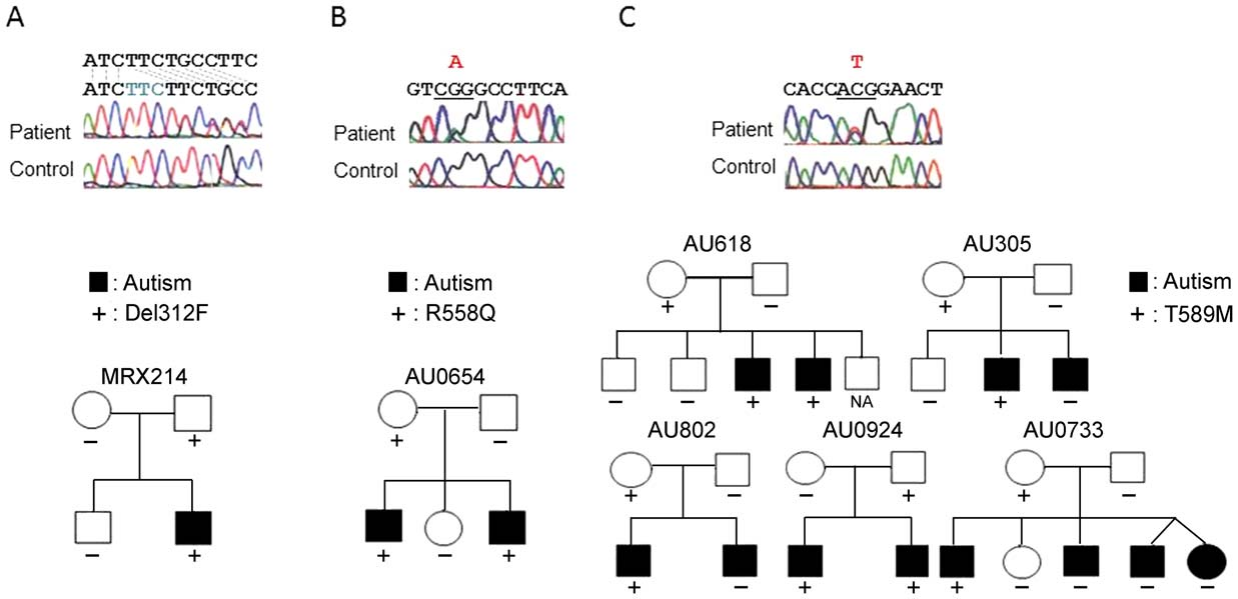
Sequences of mutations detected and family trees of the patients with each base substitution. (A) Japanese family of MRX214 with Del 312F in *GPR37*. (B) Caucasian family of AU0654 with mutation R558Q in *GPR37*. (C) Caucasian family with T589M in *GPR37*. Closed boxes and circles indicate the individual with ASD, + indicate the individual with mutations.

Two mutations were detected in the *GPR37* gene (Table 1) in patients with autism. One was 1585–1587 ttc del (Del312F), detected in a male Japanese patient. This mutation was also detected in the patient's unaffected father, but not in his unaffected mother or unaffected brother (Figure 1). The other mutation was G2324A (R558Q), detected in a male Caucasian patient. This mutation was also detected in the patient's affected brother and unaffected mother (Figure 1). The clinical features of the patients with Del312F and R558Q were autism with mild-intellectual disability. They could speak simple words and had no epileptic seizures.

**Table 1:** Results of the analysis of *GPR37*.

Base Change	AA change	Patients	Control	Report
***Japanese patients***	***(n = 72)***			
G1980C	L443L	(G/G) 14/72		SNP
		(G/C) 41/72		
		(C/C) 17/72		
**Del 1585–1587 ttc**	**Del312F**	**1/72**	**0/145**	**Fa+,Mo−,bro−**
C2058G	I469M	(C/G) 1/72	1/145	Fa−,Mo+
***Caucasian patients***	***(n = 194)***			
T699C	L16L	(T/T) 90/96		No
		(T/C) 5/96		
		(C/C) 1/96		
C1311T	A220A	(C/C) 85/96		No
		(C/T) 10/96		
		(T/T) 1/96		
T891C	F80F	(C/T) 2/190		No
C1698T	T349T	(C/C)105/190		SNP
		(T/T) 8/190		
		(T/C) 77/190		
G1980C	L443L	(G/G) 58/190		SNP
		(G/C) 94/190		
		(C/C) 38/190		
**G2324A**	**R558Q**	**(G/A) 1/194**	**0/200**	**Fa−,Mo+,bro+**
C2417T	T589M	(C/T) 5/194	1/200	

We also found other base substitutions in the *GPR37* gene, including C2058G (I469M) and C2417T (T589M) (Table 1). The I469M mutation was detected in a male Japanese patient, his unaffected mother, and one control sample. The T589M mutation was detected in seven male Caucasian patients from five families; among these families, the mutation was found in four apparently unaffected mothers and one unaffected father (Figure 1). None of the siblings that carried the variants were unaffected. However, the T589M variant was not found in 5 affected siblings among three families, including one non-identical twin. Among the Caucasian controls, one out of 200 had the variant.

In addition, we found some previously reported SNPs, including G1980C (L443L), which was detected in both the Japanese and Caucasian patients, and C1698T, which was detected in some Caucasian patients. We also detected three SNPs that had not been previously reported, including T699C (L16L), C1311T (A220A), and T891C (F80F). These SNPs did not alter the predicted amino acid sequence of GPR37.

**Figure 2:**
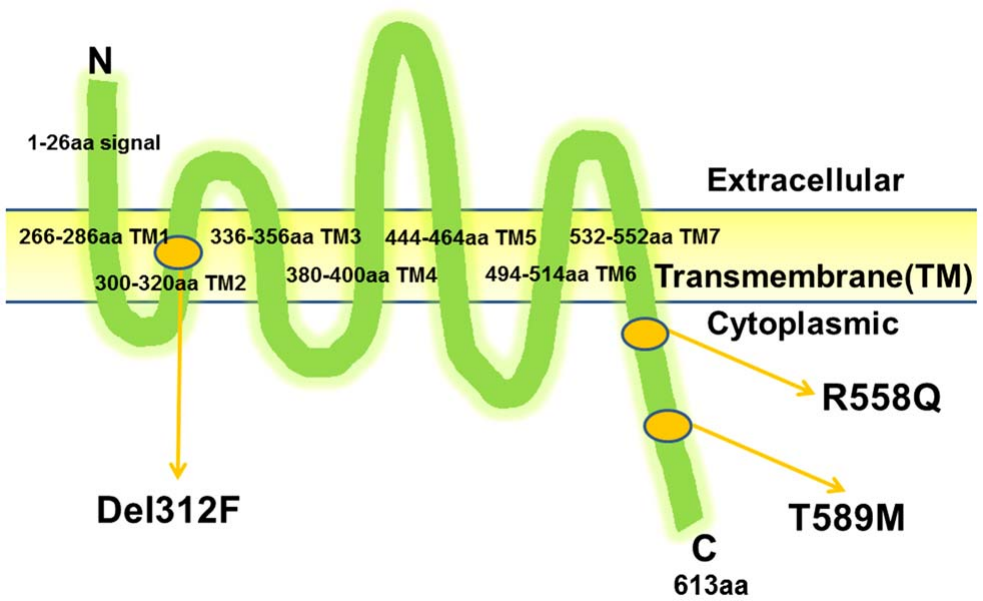
The location of the GPR37 mutations. Arrow indicates the position of Del312F, R558Q and M589T.TM, transmembrane domain.

The GPR37 consists of 614 amino acids and has seven transmembrane domains [22], [23]. GPR37 has a putative extracellular N-terminus. The homology between humans and mice is low for the putative extracellular N-terminus (62% amino acid identity), but high for the transmembrane domain and C-terminus (both 98% identical) [23]. The 558R and 589T amino acid residues of GPR37 are located just distal to the last transmembrane domain (TM, TM7) in the C-terminal region (Figure 2). These amino acids were conserved between humans and mice. However, the 469I amino acid residue is located in the cytoplasmic domain between TM5 and TM6. The deletion, Del312F, resides in the TM2, a highly conserved region.

**Figure 3:**
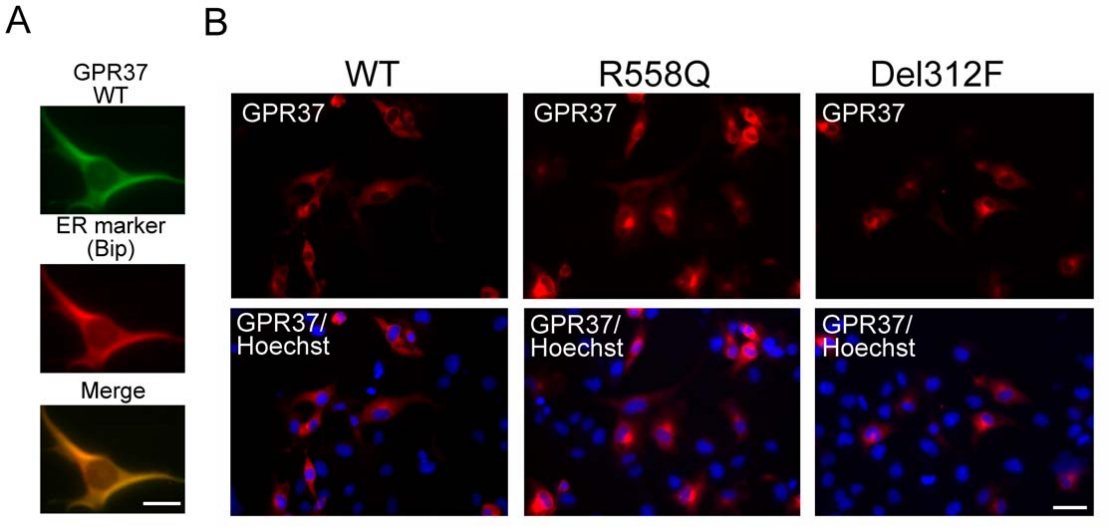
The localization of wild-type and mutated GPR37 in C2C5 cells. (A) Co-localization of wild-type GPR37-myc with endoplasmic reticulum (ER)-marker (Bip/GRP78). Scale bar: 10 µm. (B) C2C5 cells were transfected with wild-type GPR37-myc, GPR37(R558Q) -myc or GPR37(Del312F)-myc for 28 h after transfection. Cells were fixed, and immunostained with anti-myc (GPR37, red) and Hoechst (blue). Scale bar: 20 µm.

When transfected into C2C5 cells, GPR37 was sparsely co-localized with a marker of the endoplasmic reticulum (ER), Bip/glucose-regulated protein(GRP78) (Figure 3A), as previously reported [24]. We examined the intracellular localizations of wild-type and mutated GPR37 proteins in C2C5 cells (Figure 3B). Compared to the wild-type GPR37, we found high accumulations of the mutated GPR37(R558Q) and GPR37(Del312F) proteins (Figure 3B).

**Figure 4:**
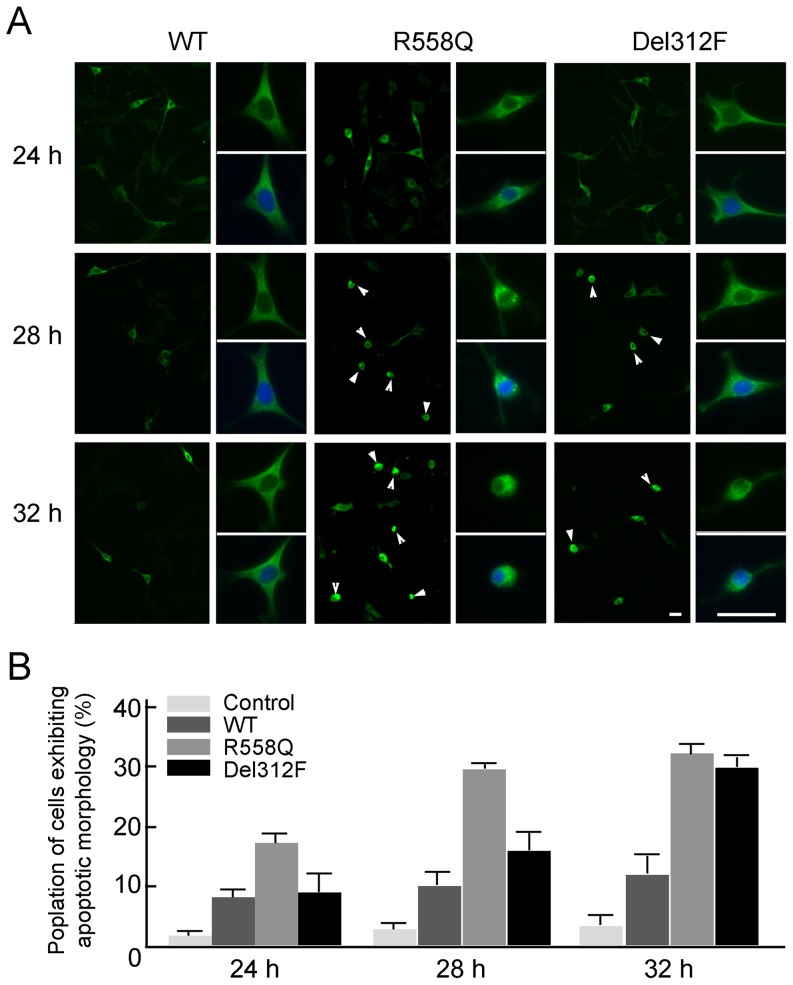
Appearance of apoptotic cells and cell having intracellular accumulation of GPR37(R558Q) and GPR37(Del312F) in time-dependent manner. (A) Apoptotic cells expressing wild-type GPR37-myc, GPR37(R558Q)-myc or GPR37(Del312F)-myc. Green, GPR37 wild-type or mutants. Blue, Hoechst. Right pictures, high magnification of left pictures. Arrowheads, apoptotic morphology. Scale bars: 40 µm. (B) The percentage of the apoptotic cells in the cells expressing, GPR37(R558Q)-myc or GPR37(Del312F)-myc. Values are mean ± standard error (SEM). All experiments were performed three times. A significant difference of the percentage of cells exhibiting apoptotic morphology was detected in the cells expressing wild-type GPR 37 and mutated GPR37. (Student's *t*-test; *p*<0.05). Control, cells expressing vacant vector (GFPC1-vector).

We examined the accumulation of GPR37(R558Q) and GPR37(Del312F) in cultured cells in a time-dependent manner (Figure 4). Compared to the wild-type GPR37, the GPR37(R558Q) and GPR37(Del312F) accumulated; in addition, these cells exhibited accelerated apoptotic morphology, with cellular and nuclear shrinkage (Figure 4A). The apoptotic cell population of cells that expressed wild-type GPR37 was about 8.3% at 24 h and 11.8% at 32 h; in contrast, the apoptotic cell population of cells that expressed GPR37(R558Q) and GPR37(Del312F) were 14.1% and 8.9% at 24 h and 31.7% and 29.1% at 32 h, respectively (Figure 4B). Furthermore, some of cells exhibiting apoptotic morphology of the cells expressing GPR37 mutants were the anti-active caspase-3 (apoptotic marker)-positive (Figure S1).

## Discussion

In our analysis of *GPR37*, we detected two mutations, the Del312F in one Japanese male patient and the R558Q in one Caucasian patient. These mutations were present in affected individuals and absent in 280 Japanese controls and 200 Caucasian controls. Del312F is localized on the second transmembrane domain in a highly conserved region. Because mutations in conserved regions are expected to cause deleterious effects, it was not surprising that Del312F was associated with the affected status. Thus, these mutated *GPR37* is likely related to ASD. Two other missense mutations, C2058G (I469M) in one Japanese patient and C2417T (T589M) in five Caucasian patients also suggested the mutation of *GPR37* was related to ASD.

The Del312F mutation was carried by an apparently unaffected father, and the R558Q mutation was carried by an unaffected mother and an affected brother. The T589M mutation was also found in unaffected parents and was not found in some of affected brothers. These inheritance patterns of the mutated *GPR37* suggested three possible hypotheses.

First, the predisposing variant may be transmitted from an unaffected parent to affected offspring in a dominant fashion with the variable penetrance, higher in males and lower in females. The presence of a mutation in apparently unaffected parents has often been observed in ASD pedigrees, although no clear explanation was reported; for example, this was also observed in ASD pedigree with the *CNTNAP2* mutation [4]. Therefore, this finding does not reduce the relevance of the mutation to the disease; rather, it may reflect a distinct pattern of ASD inheritance. An additional unrevealed gene or factors may contribute to disease effects in these families.

We have proposed the possible involvement of ER stress caused by the mutated molecules in the ASD [25]; the mutated CADM1 proteins exhibited high susceptibility to a processing enzyme, which led to the accumulation of processed fragments in the ER [26]. Accumulations of the mutated GPR37(R558Q) and GPR37(Del312F) proteins in the ER and their induced cell death, so called as ER stress cell death, suggest that not only loss-of-function but also gain-of-function of the mutated GPR37 is involved in the ASD. This hypothesis would explain the mutation in the unaffected parents if gain-of-function exhibited anticipation as shown in their children with ASD.

Second, some mutated alleles may show deleterious effects only in offspring that are susceptible to ASD, as shown in families MRX214 with Del341F and AU0654 with R558Q. Or, MRX and AU0653 may simply be families of autosomal recessive inheritance. However, inheriting the T589M allele alone would be insufficient for an individual to manifest ASD, and additional predisposing variants must be present in all five families. This hypothesis predicts that the manifesting siblings in families, AU305, AU802, and AU0733, should carry at least one predisposing variant other than T589M; loss-of-function or gain-of-function of Gpr37(T589M) may not be sufficient to manifest ASD. The presence of five pedigrees in this study strongly suggested that ASD is a multigenetic trait, at least for some types of ASD. Thus, one variant alone would not achieve the threshold for expressing ASD pathology in cells; only individuals that received additional predisposing variants would express the ASD phenotype. To identify this unrevealed variant will be one of the important issues in future study.

Third, we cannot completely rule out the possibility that we found a false positive association between T589M and ASD in 7 affected individuals. However, it is clear that this variant had participated in ASD development in these families, because this variant was detected in only one of 200 controls.

GPR37 is similar to endothelin receptors A and B [9], and it is described as a Parkin-associated endothelin-like receptor (PaelR). Moreover, GPR37 is a substrate of the Parkin enzyme, which plays a key role in Parkinson disease [27], [28]. Recently, the neuropeptide, head activator (HA), has been identified as a high affinity ligand for GPR37 [29]. HA was originally isolated from hydra, and it was found to mediate head-specific growth and differentiation. In adult mammals, HA enhances neurite outgrowth and is neuroprotective [29]. In the developing brain, HA stimulates the entry into mitosis and promotes proliferation of cells in the nervous and neuroendocrine systems [30]. Moreover, GPR37 modulates dopamine transporter activity and the behavioral response to dopaminergic drugs [31], [32]. These findings suggest the possibility that GPR37 plays a role in brain development, and a deficiency in GPR37 function underlies the pathology of ASD. Future studies that describe newly detected mutations should investigate whether they are involved in anatomical and functional deficiencies in the developing brain. *GPR37*-deficient mice showed reduced dopamine levels in the striatum, abnormal locomotor activity, and heightened sensitivity to amphetamine [21]. However, this mouse model was analyzed only from the viewpoint of Parkinson disease or depression. Further analysis of a *GPR37* knockout mouse should be performed from the viewpoint of ASD. The loss of function of GPR37 and gain-of-function of the mutated GPR37 may be a crucial molecule for Parkinson disease and ASD, respectively. Dopamine-dependent neurons may be also involved in the ASD.

The present study described mutations in the *GPCR* gene were associated with the manifestation of ASD; some appeared to have a causative effect, and others appeared to confer susceptibility to ASD under predisposing conditions. The presence of unaffected family members that carried the mutation suggested that the manifestation of ASD was also influenced by other factors.

## Materials and Methods

### Patients

Lymphocytes were obtained from 72 unrelated Japanese ASD patients including those with autism, pervasive developmental disorder not otherwise specified and Asperger syndrome, who lived in Tochigi and Ibaragi Prefecture and visited Jichi Medical School Hospital. Their conditions were diagnosed according to the DSM-IV criteria.

There were 57 male and 15 female patients. The age of the patients ranged from 3 to 23 years. The IQ and language ability of the patients varied from severely affected to normal. Four patients had siblings who also suffered from ASD, and others were sporadic. Control samples were obtained from normal Japanese adult volunteers after obtaining their written informed consent. We also obtained the DNA of 200 Caucasian patients from the Autism Genetic Resource Exchange (AGRE) Consortium (Cure Autism Now, Los Angeles, CA). AGRE samples were 172 males and 28 females, and all were familial cases. They included ASD and PDD. Caucasian control samples were obtained from Coriell Institute (Camden, New Jersey).

### Ethics Statement

Written informed consent was obtained from the parents of all subjects, and the study was approved by the Bioethics Committee for Human Gene Analysis of Jichi Medical University (approval number, 11–25).

### PCR Amplification

Genomic DNA was extracted from the peripheral blood lymphocytes or lymphoblasts using the standard method following the manufacture's instructions. All exons and its boundaries of *GPR37* were amplified separately by PCR. The primer sequences of *GPR37* were listed in Table 2. PCR was performed in a 50 µl reaction volume containing 10xPCR Buffer, 200 µM dNTPs, 100 ng genomic DNA, 0.5 µM each primer and 0.25 U Taq DNA polymerase (Takara, Tokyo, Japan). PCR were carried out in the GeneAmp PCR system 9700 (Applied Biosystems, Foster city, CA) by denaturing for 5 min at 94°C, then 35 cycles of 30 sec at 94°C, 30 sec at annealing temperature of each primer (Table 2), and 30 s at 72°C, followed by a final extension at 72°C for 7 min.

**Table 2:** Primer sequences of *GPR37*.

Primer sequence	Products size (bp)
1F ATGTGCCTAACTCTAGCAGC	285
1R GTCTCTTGCACAATTTCCCG	
2F GAAACGAAACTTGTCTGGGG	339
2R TCTGAAGGAAGAGCTGGAGG	
3F CTTCTGAAACTTTGGGGAG	415
3R ACAGACACATGACCGCGTAG	
4F ATCCTTGGGTGAAGGAATCC	431
4R CCAGCAGCAGTTCTCTGATG	
5F GGACACCGGGAATAAATGTC	443
5R ACAGGCTTTCTCTGCTTTGC	
6F CCTGCTCTCTAGTGACTGC	517
6R CACGGGATATGAAAATCAAAC	

### DNA construction and site-directed mutagenesis

The full-length of GPR37 cDNA was isolated from human cDNA library (Stratagene, La Jolla, CA). The enzyme-digested PCR DNA fragments corresponding to full-length GPR37, and mutated GPR37 were subcloned into pGEM-T easy vector (Promega, Madison, WI), and then the pcDNA4/TO/myc-His expression vector (Invitrogen, CA), and transfected into C2C5 cells [33] using the calcium-phosphate method.

To generate GPR37 mutants, site-directed mutagenesis were performed by using QuikChange II Site-Directed Mutagenesis kit (Stratagene) with following primers: for mutation of R558Q, sense primer; 5′-AAACCCTTCAGTCAGGCCTTCATGGAGT-3′, antisense primer; 5′-ACTCCATGAAGGCCTGACTGAAGGGTTT-3′, for mutation of del312F, sense primer; 5′-TTTCTCATCATCTTCTGCCTTCCGCTGGTCAT-3, NLS1 antisense primer; 5′-ATGACCAGCGGAAGGCAGAAGATGATGAGAAA-3. Underlined nucleotides were mutated sites. Mutations were confirmed by DNA sequencing. The experiments were approved by Genetic Modification Safety Committee of Jichi University (approval number, 11–52), and were carried out under the jurisdiction of the Ministry of Education, Culture, Sports, Science and Technology.

### Immunostaining

Transfected cells (vacant vector, GPR37 wild-type and mutants) were fixed in 4% Paraformaldehyde in PBS, respectively. After they were incubated with blocking reagent (PBS containing 1% goat serum, 1% skim milk and 0.1% Triton X-100) for 1 h at room temperature, they were then subjected to the immunostaining using mouse anti-myc (Nacalai, Kyoto, Japan), rabbit anti-Bip/GRP78 (Stressgen, Kampenhout, Belgium), and anti-active caspase-3 [34]. Alexa Fluor 488 and Alexa Fluor 568 conjugated secondary antibodies against mouse and rabbit IgG were purchased from Molecular Probes (Eugene, OR). Nuclei were detected by Hoechst 33342 (Molecular Probes). Immunofluorescence was viewed using a confocal laser-scanning microscope (CSU-10, Yokogawa Electric Co., Tokyo, Japan). Percentages of cells showing apoptotic-morphology were determined by counting 200 cells expressing GPR37 wild-type and mutants at 24 h–32 h after transfection. The values are averages of the percentages of the number of cells obtained in three experiments. Results were analyzed using student's *t*-tests (*p*<0.05 was considered statistically significant).

## Supporting Information

Figure S1 **Appearance of apoptotic cells and cell having intracellular accumulation of GPR37(R558Q) and GPR37(Del312F) at 28 h after transfection.** (A) Apoptotic cells expressing wild-type GPR37-myc, GPR37(R558Q)-myc or GPR37(Del312F)-myc. Arrowheads, anti-active caspase-3 positive cells(red) that expressed wild-type and mutated GPR37 (green). Blue, Hoechst. Scale bar: 40 µm. (B), high magnification of (A). Scale bar: 25 µm.
(TIF)
