# Supplementary figures and images for: Mutation in Parkinson Disease-Associated, G-Protein-Coupled Receptor 37 (GPR37/PaelR) Is Related to Autism Spectrum Disorder
Source: PLoS One. 2012 Dec 12;7(12):e51155. doi: 10.1371/journal.pone.0051155 (PMC3520984; doi:10.1371/journal.pone.0051155)

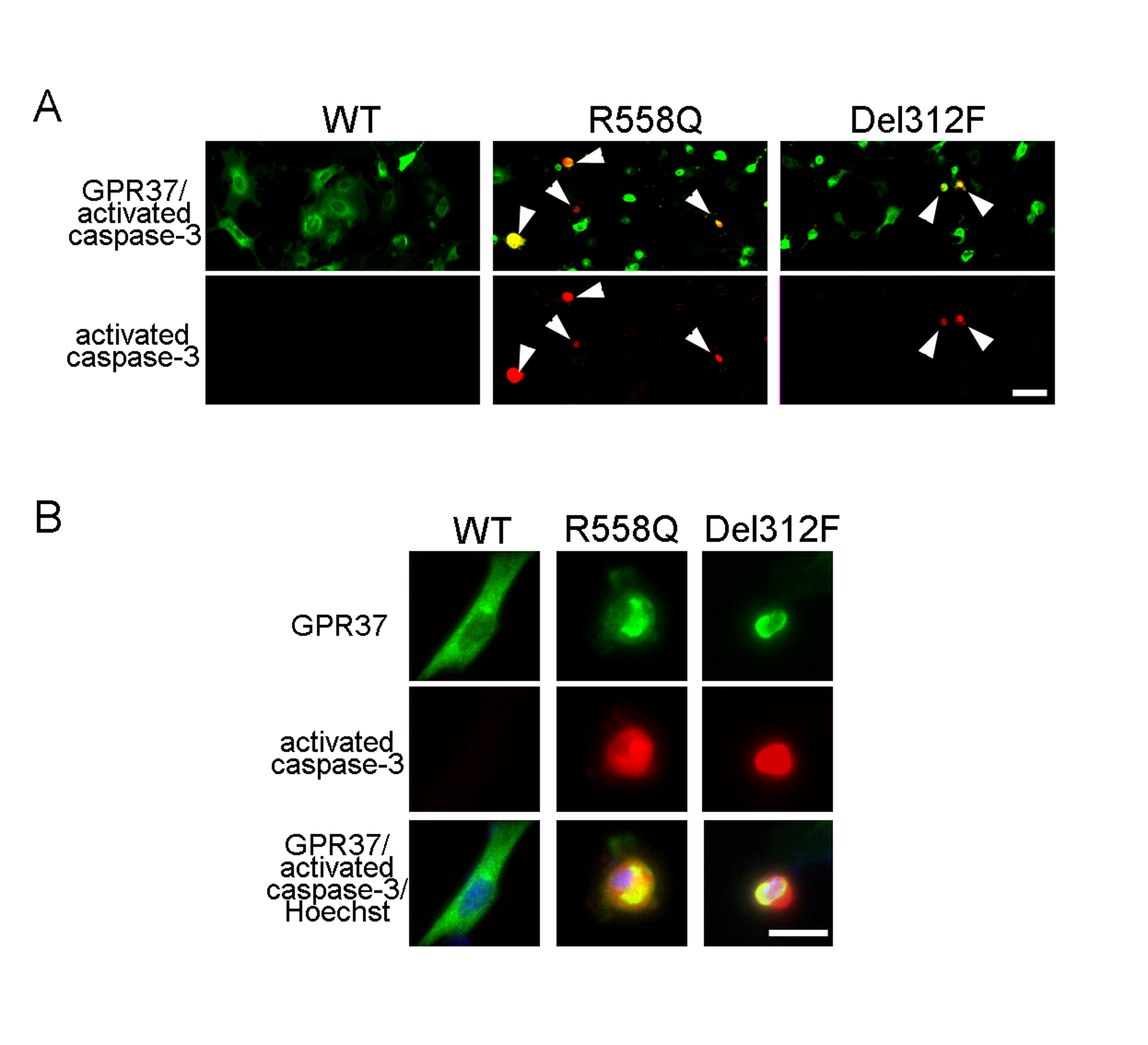

Supplement: Figure S1 — Appearance of apoptotic cells and cell having intracellular accumulation of GPR37(R558Q) and GPR37(Del312F) at 28 h after transfection. (A) Apoptotic cells expressing wild-type GPR37-myc, GPR37(R558Q)-myc or GPR37(Del312F)-myc. Arrowheads, anti-active caspase-3 positive cells(red) that expressed wild-type and mutated GPR37 (green). Blue, Hoechst. Scale bar: 40 µm. (B), high magnification of (A). Scale bar: 25 µm. (TIF) [file pone.0051155.s001.tif]
